# Tau PET burden in Brodmann areas 35 and 36 is associated with individual differences in cognition in non-demented older adults

**DOI:** 10.3389/fnagi.2023.1272946

**Published:** 2023-12-14

**Authors:** Nisha Rani, Kylie H. Alm, Caitlin A. Corona-Long, Caroline L. Speck, Anja Soldan, Corinne Pettigrew, Yuxin Zhu, Marilyn Albert, Arnold Bakker

**Affiliations:** ^1^Department of Psychiatry and Behavioral Sciences, Johns Hopkins University School of Medicine, Baltimore, MD, United States; ^2^Department of Neurology, Johns Hopkins University School of Medicine, Baltimore, MD, United States

**Keywords:** ^18^F-MK6240 Tau PET, entorhinal cortex, mild cognitive impairment, episodic memory, BA35, BA36

## Abstract

**Introduction:**

The accumulation of neurofibrillary tau tangles, a neuropathological hallmark of Alzheimer’s disease (AD), occurs in medial temporal lobe (MTL) regions early in the disease process, with some of the earliest deposits localized to subregions of the entorhinal cortex. Although functional specialization of entorhinal cortex subregions has been reported, few studies have considered functional associations with localized tau accumulation.

**Methods:**

In this study, stepwise linear regressions were used to examine the contributions of regional tau burden in specific MTL subregions, as measured by ^18^F-MK6240 PET, to individual variability in cognition. Dependent measures of interest included the Clinical Dementia Rating Sum of Boxes (CDR-SB), Mini Mental State Examination (MMSE), and composite scores of delayed episodic memory and language. Other model variables included age, sex, education, APOE4 status, and global amyloid burden, indexed by ^11^C-PiB.

**Results:**

Tau burden in right Brodmann area 35 (BA35), left and right Brodmann area 36 (BA36), and age each uniquely contributed to the proportion of explained variance in CDR-SB scores, while right BA36 and age were also significant predictors of MMSE scores, and right BA36 was significantly associated with delayed episodic memory performance. Tau burden in both left and right BA36, along with education, uniquely contributed to the proportion of explained variance in language composite scores. Importantly, the addition of more inclusive ROIs, encompassing less granular segmentation of the entorhinal cortex, did not significantly contribute to explained variance in cognition across any of the models.

**Discussion:**

These findings suggest that the ability to quantify tau burden in more refined MTL subregions may better account for individual differences in cognition, which may improve the identification of non-demented older adults who are on a trajectory of decline due to AD.

## 1 Introduction

There is considerable evidence that the pathological hallmarks of Alzheimer’s disease (AD) emerge many years before clinical symptoms of mild cognitive impairment (MCI; Sperling et al., 2011; Pletnikova et al., 2018). Amyloid (Aβ) plaques and neurofibrillary tau tangles, the neuropathological hallmarks of AD, have been shown to accumulate among cognitively normal (CN) older adults, with the percentage of CN individuals showing these pathological changes varying with age (Brier et al., 2016). Biomarker evidence of these pathological changes among asymptomatic individuals is reflected in studies involving the assessment of cerebrospinal fluid (CSF; Blennow et al., 2010) and positron emission tomography (PET; Bao et al., 2017). Importantly, PET studies can provide details about the spatial distribution of amyloid and tau in the brain during the early phases of AD.

While the distribution of amyloid deposition in the brain early in the course of disease does not appear to be particularly informative about the likelihood of progression from normal cognition to MCI (Scheinin et al., 2009), tau tends to deposit in a systematic fashion early in the course of AD and then spreads in a relatively predictable manner to other brain regions (Pascoal et al., 2018). Post-mortem studies have provided valuable insights into the spatial and temporal progression of tau pathology, revealing that the formation of neurofibrillary tangles in the initial stages occurs in the entorhinal cortex (EC), specifically in the transentorhinal cortex (TEC), which serves as the transition between the lateral portions of the EC and the perirhinal cortex (Braak and Braak, 1990; Braak et al., 2006; Kaufman et al., 2018). Notably, tau accumulation in the TEC is common by age 60, even among CN older adults and in the absence of concurrent Aβ pathology (Maass et al., 2018). Tau pathology then continues to spread through other medial temporal lobe (MTL) regions (Braak et al., 2006), which are of critical interest given their well-established roles in episodic memory functioning (Dickerson and Eichenbaum, 2010). Moreover, tau deposition has been observed to exhibit a more consistent and robust association with cognitive decline throughout aging and the AD spectrum (Nelson et al., 2012; Brier et al., 2016; Ossenkoppele et al., 2020), compared to amyloid deposition.

Fortunately, tau-specific PET tracers permit the investigation of regional associations between tau pathology and cognition *in vivo* (Johnson et al., 2016; Ossenkoppele et al., 2016, 2020; Chiotis et al., 2021). Over the past decade, numerous PET radiotracers have been introduced to visualize tau pathology deposition *in vivo* (Ossenkoppele et al., 2016, 2021; Hall et al., 2017; Ricci et al., 2021). Compared to initial tracers, ^18^F-MK6240 (Malarte et al., 2021), a second-generation tau PET tracer, has demonstrated favorable imaging characteristics and spatial distributions consistent with the spread of neurofibrillary tangles reported in autopsy tissue (Pascoal et al., 2018; Lohith et al., 2019; Betthauser et al., 2020). Importantly, ^18^F-MK6240 exhibits subnanomolar affinity for tau tangles, with a dissociation constant of approximately 0.3 nM, making it an improved tau radioligand, with minimal binding to off-target sites in the basal ganglia and choroid plexus (Hostetler et al., 2016). Comparative studies with ^18^F-AV1451 PET have shown that ^18^F-MK6240 offers a higher dynamic range of standardized uptake value ratio (SUVR) values across different Braak stages in AD patients, indicating its potential for enhanced sensitivity for early detection and monitoring of AD progression (Gogola et al., 2022; Krishnadas et al., 2023).

The high binding specificity of ^18^F-MK6240 PET imaging provides an opportunity to investigate the relationship between tau burden in specific MTL subregions and individual differences in cognitive functioning among older adults. For example, recent studies in rodents have shown that the lateral EC and perirhinal cortex support encoding of object/content information, while medial EC and parahippocampal regions facilitate encoding of context and spatial information (Knierim et al., 2014). Functional specialization in these regions also appears differentially impacted by aging and AD, with more lateral EC-dependent functions tending to be preferentially impacted by aging (Reagh et al., 2018; Tran et al., 2021, 2022). Since both AD-related tau pathology and age-related functional changes relate to MTL structures non-uniformly, subregion measurements may be more sensitive to early disease changes, compared to currently employed segmentation approaches.

Therefore, the primary objective of this study was to examine the associations between tau accumulation in MTL subregions and individual differences in cognition in a cohort of non-demented older adults. Utilizing detailed segmentation of MTL subregions with advanced registration approaches, this study examined whether quantifying tau burden in smaller MTL subregions accounts for a greater proportion of the variance in cognitive performance when compared to larger MTL areas utilized by traditional PET image analysis approaches. A total of 8 regions of interest (ROIs) were selected *a priori* based on the location of post-mortem tau accumulation in the early stage of AD (Braak and Braak, 1990; Braak et al., 2006). These regions included the left and right EC obtained from FreeSurfer parcellations (Fischl, 2012), as well as the left and right entorhinal cortex (labeled ERC to distinguish the different softwares), Brodmann area 35 (BA35), and Brodmann area 36 (BA36) obtained from the Automated Segmentation of Hipppocampal Subfields (ASHS) software (Xie et al., 2019). FreeSurfer EC parcellations encompass the anterior portion of the parahippocampal gyrus, including the medial bank of the collateral sulcus, as well as a portion of the TEC (Braak and Braak, 1991; Taylor and Probst, 2008). ASHS BA35 largely overlaps with TEC (Braak and Braak, 1991; Braak et al., 2006) and a portion of the perirhinal cortex, while BA36 predominantly encompasses the perirhinal cortex (Xie et al., 2019). These segmentations were applied to imaging data obtained from the Biomarkers of Cognitive Decline Among Normal Individuals (BIOCARD) study that has detailed clinical and cognitive evaluations of the participants, as well as extensive biomarker data (Albert et al., 2014).

## 2 Methods

### 2.1 Participants

The present investigation utilized data from a subset of participants enrolled in the BIOCARD study, which is an ongoing longitudinal prospective cohort study aimed at understanding the early phases of AD. The BIOCARD study began in 1995 at the intramural program of the National Institutes of Health (NIH) and continued at Johns Hopkins University (JHU) starting in 2009. All participants were cognitively normal when enrolled and, by design, 75% had a family history of dementia. When the study was conducted at the NIH, clinical and cognitive assessments were completed annually and CSF, blood, and magnetic resonance imaging (MRI) scans were collected approximately every other year. In 2015, biennial collection of MRI, CSF, and amyloid PET data commenced, and tau PET imaging data collection using ^18^F-MK6240 began in 2020. The participants were primarily middle-aged (*M* = 57.3, *SD* = 10.4, range = 20.0–85.8) at enrollment. For further information on the BIOCARD cohort, see Albert et al. (2014).

The current study sample included all participants from the BIOCARD study who underwent both a ^18^F-MK6240 tau PET scan and an amyloid PET scan using ^11^C-PiB. All included individuals also had a concurrent T1-MPRAGE scan and cognitive testing obtained during the same visit. The cognitive status of each participant in the study sample was determined by a consensus diagnosis from the JHU BIOCARD Clinical Core staff following the study visit. The diagnostic approach adheres to the guidelines of the National Institute on Aging – Alzheimer’s Association working group and is comparable to the approach used at the National Institute on Aging Alzheimer’s Disease Centers program (Albert et al., 2011; McKhann et al., 2011). First, a syndromic diagnosis is established based on three sources of information, including (1) clinical data on the individual’s medical, neurological, and psychiatric status; (2) reports of cognitive changes from the participant and their informants; and (3) evidence of cognitive performance decline based on review of longitudinal neuropsychological assessments of multiple cognitive domains with comparison to published norms. Next, for participants with cognitive impairment, the likely syndromic etiology was determined based on the available neurologic, medical, and psychiatric information; more than one etiology could be endorsed. All diagnoses were made without knowledge of the imaging or fluid biomarker data.

The resulting study sample consisted of 93 participants, including 82 CN individuals and 11 participants with a diagnosis of MCI.

### 2.2 Clinical and cognitive assessments

The clinical assessment of the participants included the administration of the Clinical Dementia Rating Scale (CDR; Morris, 1993). The cognitive assessment included a battery of standardized neuropsychological tests covering various cognitive domains, including memory, executive function, language, visuospatial ability, attention, speed of processing, and psychomotor speed. A detailed description of these tests has been previously published (Albert et al., 2014). Our analysis focused on several key measures, including the Clinical Dementia Rating Sum of Boxes (CDR-SB) score, the Mini-Mental State Examination (MMSE) score, as well as composite scores for delayed episodic memory and language. Briefly, the language composite score was established using confirmatory factor analysis (Soldan et al., 2019) and was based on three tests: Boston Naming Test, Category Fluency (animals), and Letter Fluency (FAS). For each of these tests, scores were z-transformed and weighted by their respective standardized factor loadings from a confirmatory factor analysis. The resulting transformed scores were averaged to obtain language composite scores for each subject [see Soldan et al. (2019) for further details]. The delayed episodic memory composite score [previously described by Alm et al. (2022)] was based on the California Verbal Learning Test (CVLT) long delay free recall and the Wechsler Memory Scale Logical Memory (LM) delayed recall. Task scores were z-transformed, giving equal weight to each memory measure, and transformed scores were then averaged to obtain delayed episodic memory composite scores for each subject.

### 2.3 MRI imaging

T1-weighted MRI images were acquired using a magnetization-prepared rapid acquisition with gradient echo (MPRAGE) sequence on a 3 T MRI scanner with a 32-channel head coil (Philips Achieva, Eindhoven, Netherlands) to establish anatomical reference for PET image registration. The following parameters were employed: repetition time (TR)/echo time (TE) = 6.8 ms/3.1 ms, shot interval = 3,000 ms, inversion time = 843 ms, flip angle = 8°, field of view (FOV) = 256 mm × 256 mm with 1.0 × 1.0 × 1.2 mm^3^ voxels and 170 sagittal slices. Prior to image processing, a visual quality control assessment was conducted to ensure the integrity and suitability of the acquired images.

### 2.4 Amyloid and tau PET imaging

PET scans were acquired using a GE DISCOVERY RX PET/computed tomography (CT) scanner in 3D acquisition mode. Tau tangle ^18^F-MK6240 PET images were acquired at 90 min ± 1 min after a single bolus intravenous injection of 5.0 mCi ± 10% mCi (volume ≤ 10 mL) of the radiotracer followed by a 10 to 20 mL saline flush. ^11^C-PiB was used for amyloid PET imaging, and images were acquired in a 20-min brain scan session 50 min ± 1 min after injection of 15 mCi ± 1.5 mCi of radiotracer. Syringes were used to measure the residual activity post-injection.

The PET images were reconstructed into six frames for ^18^F-MK6240 and four frames for ^11^C-PiB using the three-dimensional ordinary Poisson ordered-subset expectation maximization (3D OP-OSEM) algorithm with corrections applied for detector efficiency, decay, dead time, attenuation, and scatter (Hudson and Larkin, 1994). Each PET data frame was evaluated to verify adequate count statistics and absence of head motion. Low-dose CT (120 KeV, 80 mA; Slice thickness 3.75 mm; Slice separation 3.3 mm; FOV 500 mm) based attenuation correction (STANDARD Kernel) was applied. The final PET images were reconstructed into 2 × 2 × 3.27 mm^3^ voxels in units of radioactivity concentrations (Bq/ml).

To generate Standard Uptake Value (SUV, g/mL) PET images, the Bq/ml PET images were normalized to the patient’s body weight (BWt) and the injected dose (ID). The SUVs were calculated as the mean radioactivity per injected dose per weight using the formula SUV = A/(ID x BWt), where A represents the activity concentration of the PET image in Bq/mL, BWt is the patient’s body weight in grams, and ID is the injected dose in Bq. To account for radioactive decay, all SUVs were corrected based on the specific half-life of the F-18 radionuclide (for ^18^F-MK6240 PET) and C-11 radionuclide (for ^11^C-PiB PET). Participants were instructed to remain still for the total duration of each PET scan. ^11^C-PiB PET data were missing for 2 CN participants. Representative ^18^F-MK6240 PET image examples are shown in Supplementary Figure S1.

### 2.5 PET image analysis

Both ^18^F-MK6240 and ^11^C-PiB PET images were motion-corrected using rigid body linear registration with six degrees of freedom implemented in FSL [FMRIB (Oxford Centre for Functional MRI of the Brain) Software Library (Jenkinson et al., 2002)]. A mean PET image was calculated for each subject from the motion-corrected time series, and registered and resampled to the subject’s skull-stripped T1-weighted image using FreeSurfer (Dale et al., 1999; Greve and Fischl, 2009). To correct for partial volume effects (PVE) resulting from the limited spatial resolution of the PET scan, partial volume correction (PVC) was performed using the geometric transfer matrix (GTM) method using tools integrated into PETSurfer (Rousset et al., 1998). The GTM method assumes that within a specific ROI, the tissue is homogeneous. Because age-related changes may affect tissue homogeneity, particularly within our ROIs, the GTM method was combined with the region-based voxel-wise (RBV) approach, enabling voxel-wise PVE corrections (Thomas et al., 2011; Shidahara et al., 2017). The RBV correction relies on anatomical parcellation and an accurate point-spread function (PSF) estimation. In our PET data analysis, we employed a gaussian kernel with an isotropic full-width at half-maximum (FWHM) of 4.0 mm for the GTM-RBV-based PVC, which accounted for spill-over effects between different compartments during voxel-based correction. The T1-weighted MRI images were warped into Montreal Neurological Institute (MNI) space using the Advanced Normalization Tools (ANTS) software package (Avants et al., 2014; Tustison et al., 2014). The resulting transformation matrix from this registration was applied to each subject’s PVC-corrected PET images for normalization to MNI space. A diagram summarizing PET image analysis steps is displayed in Supplementary Figure S2.

^18^F-MK6240 PET images underwent intensity normalization by utilizing the average uptake in the pons derived from the FreeSurfer parcellation as the reference region. The choice of pons as a reference region was based on its lower variability and lower extracerebral contamination in CN and cognitively impaired subjects (Fu et al., 2023). The ^18^F-MK6240 SUVR PET images in MNI template space were utilized to generate mean SUVR values to quantify tau accumulation in subregions within the MTL. The mean ^11^C-PiB PET SUV images were converted into SUVR images using whole cerebellum gray matter defined by the FreeSurfer-derived MRI parcellations eroded by 3-voxels in 3D as the reference region. The choice of cerebellar gray matter as the reference region for ^11^C-PiB was based on its known minimal susceptibility to fibrillar amyloid deposition in AD, making it a reliable estimator of nonspecific PiB binding (Klunk et al., 2004; Price et al., 2005; Liu et al., 2015). The ^11^C-PiB SUVR PET images in MNI template space were used to generate mean SUVR values for amyloid-β deposition.

### 2.6 Image segmentation

The FreeSurfer image analysis pipeline (version 7.2.0) was employed for cortical reconstruction and volumetric segmentation of MPRAGE images, applying intensity normalization, skull stripping, cortical surface extraction, volumetric segmentation, and surface-based registration. Following reconstruction, the datasets were visually reviewed for segmentation accuracy and any errors were corrected (Dale et al., 1999; Fischl et al., 1999, 2002; Ségonne et al., 2007; Fischl, 2012).

Segmentation of additional MTL subregions was subsequently completed using the ASHS software, using the ASHS-T1 atlas specifically designed for older adults (also known as ASHS-PMC-T1 atlas; Yushkevich et al., 2014, 2016; Xie et al., 2019). Briefly, ASHS-T1 employs a series of steps to accomplish the automatic segmentation task. First, the target MRI scan is up-sampled to a resolution of 0.5×0.5×1.0 mm^3^ using a non-local-mean super-resolution algorithm (Manjon et al., 2010). Then, symmetric greedy diffeomorphic registration within the ANTs software (Avants et al., 2008) is used to warp each segmentation atlas to the target MRI scan. A joint label fusion algorithm combines the anatomical labels from the warped atlases, generating a consensus segmentation. This fusion process assigns spatially varying weights to each atlas based on patch-level similarity to the target image, while considering potential redundancy among the atlases (Wang et al., 2013). Additionally, a corrective learning algorithm corrects systematic segmentation biases using classifiers trained from leave-one-out segmentation of the atlas images (Wang et al., 2011). Bootstrapping is applied, leveraging the results of multi-atlas segmentation to improve the matching between the atlas and target images. The ASHS-generated ROIs were subsequently down-sampled using FSL FLIRT (Jenkinson and Smith, 2001; Jenkinson et al., 2002) to match the original MNI image resolution of 1.0×1.0×1.0 mm^3^. Thresholding and binarizing were accomplished using fslmaths, using a threshold >0.9. This down-sampling facilitated the extraction of SUVR measurements from the MNI-resampled tau PET image within the created MTL ROIs.

### 2.7 SUVR computation

**Figure 1:**
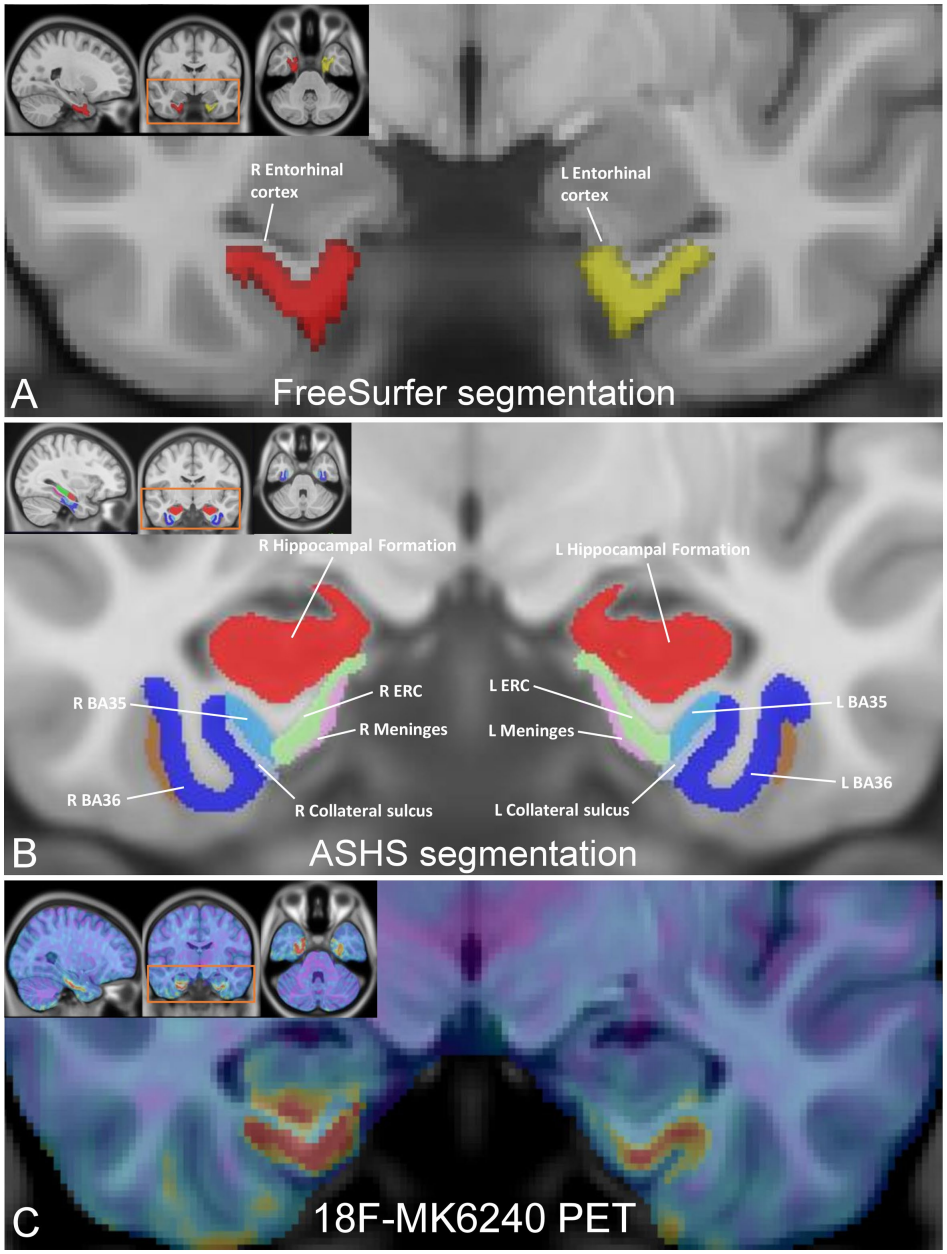
Medial temporal lobe (MTL) subregion segmentation and tau PET image registration. **(A)** FreeSurfer parcellations of the entorhinal cortex, resampled in MNI space (inset), with enlarged coronal view image highlighting the left EC (in yellow; volume 2079 mm^3^) and right EC (in red; volume 1859 mm^3^). **(B)** MNI template images (inset) displaying the segmentation of the MTL subfields using ASHS, with enlarged coronal view image showing the left/right ERC (in green; volume 566.8 mm^3^/volume 609.5 mm^3^), left/right Brodmann area 35 (in light blue; volume 623.8 mm^3^/volume 688 mm^3^), and left/right Brodmann area 36 (in blue; volume 2,443 mm^3^/volume 2,466 mm^3^). **(C)** ^18^F-MK6240 PET image (inset), registered to the MNI template with a magnified coronal view image highlighting registration between the images showing tau deposition in the ERC, BA35, and BA36 in a representative CN subject.

Mean SUVR for ^18^F-MK6240 was computed in eight ROIs, which included the left and right EC obtained from FreeSurfer parcellations, as well as the left and right ERC, BA35, and BA36 regions derived from ASHS segmentations (Figure 1). The FreeSurfer-defined EC encompasses the anterior portion of the parahippocampal gyrus, including the medial bank of the collateral sulcus, which may also include the transentorhinal region (Braak and Braak, 1991; Taylor and Probst, 2008) corresponding to BA35 or to the medial perirhinal cortex. The FreeSurfer-derived label was obtained from each participant’s native space T1-MPRAGE image and projected to the tau PET images in MNI space. The ASHS labels included ERC, BA35, and BA36, applied to the MNI-transformed ^18^F-MK6240 PET data. The utilization of the MNI common template space facilitated a direct comparison of parcellation methods. SUVR values were extracted without smoothing to preserve the highest possible resolution of the PET data.

To obtain mean SUVR values for ^11^C-PiB PET, a standardized set of ROIs obtained from FreeSurfer was defined on each hemisphere, including the precuneus, frontal, orbitofrontal, parietal, temporal, anterior cingulate, posterior cingulate, middle temporal cortices, and the global cerebral cortex. A pre-established threshold of ^11^C-PiB SUVR >1.50 was applied to determine amyloid-β positivity, as established in previous studies (Rowe et al., 2010). This threshold value served as an indicator of elevated amyloid-β deposition in the brain.

### 2.8 Statistical analysis

Statistical analyses were performed using SPSS (Version 28). Stepwise linear regressions were used to examine the contributions of regional tau burden to individual differences in CDR-SB, MMSE, and composite scores of delayed episodic memory and language. Separate regressions were constructed for each dependent variable of interest. Independent variables included age, sex, education, APOE4 status, global cerebral cortex amyloid SUVR, FreeSurfer-derived EC tau SUVR, as well as ASHS-derived ERC, BA35, and BA36 tau SUVR. Right and left hemisphere ROIs were examined in separate regressions to account for potential hemispheric differences. This approach was motivated by the statistically significant variations observed between the left and right FreeSurfer-derived EC tau SUVR values [*t* (92) = −3.22, *p* < 0.002]. Stepwise regression utilizes a mathematically driven approach to variable entry, whereby an algorithm determines which set of variables maximizes the overall proportion of explained variance. Independent variables were entered into the model one at a time and subsequently removed if they did not statistically improve the overall model. This allowed us to examine which combination of measures predicted the highest proportion of explained variance in each of the dependent variables.

Based on the results of the stepwise regressions, we performed secondary analyses using hierarchical linear regression. Unlike stepwise models, hierarchical regression allows for user-determined order of variable entry. For each dependent variable of interest, we constructed a hierarchical regression model utilizing the significant demographic predictors that emerged from the stepwise model, and then additionally entered the ASHS-derived ROIs, followed by the FreeSurfer-derived EC ROI, allowing for a direct comparison of the proportion of variance explained by the ASHS-derived versus FreeSurfer-derived labels.

## 3 Results

**Figure 2:**
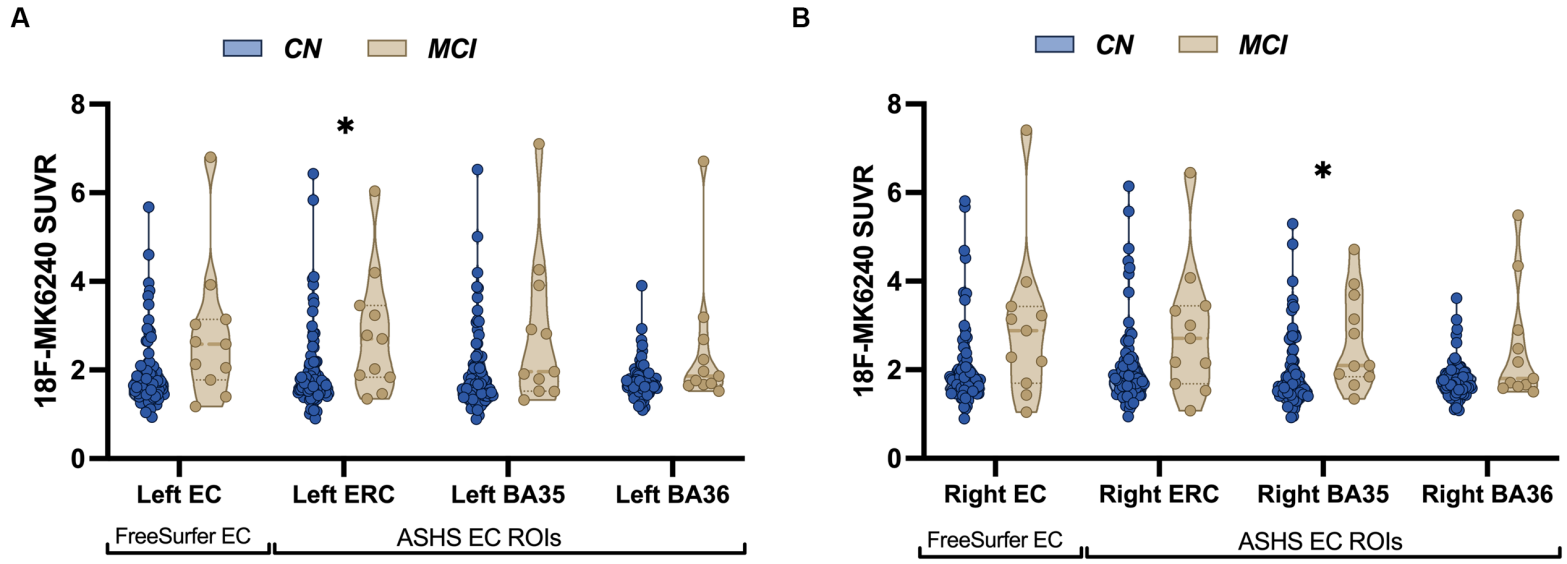
Patients with MCI show increased tau accumulation compared to control participants. Violin plots illustrating average distribution of ^18^F-MK6240 SUVR in both FreeSurfer-derived EC and ASHS-derived MTL subregions (ERC, BA35, BA36) in cognitively normal (CN) older adults and MCI individuals in the left hemisphere **(A)** and the right hemisphere **(B)**. Patients with MCI showed significantly increased tau accumulation in the left ASHS-derived ERC and right ASHS-derived BA35. The solid bars positioned in the center represent the median values, and the dotted bars indicate the interquartile range.

The sample consisted of 82 CN individuals with a mean age of 68.33 ± 9.03 years (range: 49–88 years) and 11 individuals with MCI with a mean age of 77.18 ± 6.15 years (range: 68–87 years). Sample characteristics for all participants are summarized in Table 1. Sex distribution was relatively balanced between the groups [𝜒^2^ (1) = 0.004, *p* = 0.95]. A significant group difference was observed for age [*t* (91) = −3.15, *p* = 0.002], such that the MCI patients were, on average, older than the CN participants. Group differences were also observed across all cognitive measures, with MCI patients exhibiting lower performance compared to CN participants. The proportion of individuals carrying the APOE4 allele was comparable between the two diagnostic groups [*𝜒*^2^ (1) = 0.06, *p* = 0.81], as was the distribution of amyloid positivity across groups [𝜒^2^ (1) = 0.88, *p* = 0.35]. Among the CN individuals, 25 were classified as amyloid positive and 55 as amyloid negative based on ^11^C-PiB SUVR in the cerebral cortex. Among the MCI individuals, 5 were amyloid positive and 6 were amyloid negative. Group differences in tau burden in specific MTL subfields were only significant in two subfields, left ASHS-derived ERC [*t* (91) = −2.56, *p* = 0.01] and right ASHS-derived BA35 [*t* (91) = −2.73, *p* = 0.008], with MCI patients exhibiting elevated SUVR values compared to CN (Figure 2), although this analysis was likely underpowered due to the small sample size in the MCI group.

**Table 1:** Participant characteristics.

Characteristic	Cognitively Normal (*n* = 82)	MCI (*n* = 11)	*p value*
Age, mean (SD) [range], y	68.33 (9.03) [49–88]	77.18 (6.15) [68–87]	**0.002**
Sex, No. (%)			
Men	29 (35)	4 (36)	
Women	53 (65)	7 (64)	
Education, mean (SD), y	17.19 (2.18)	16.55 (2.98)	0.38
CDR-SB, mean (SD)	0.02 (0.12)	2.00 (1.34)	**< 0.001**
MMSE score, mean (SD)	29.11 (0.93)	26.82 (2.18)	**0.006**
Logical memory (delayed), mean (SD)	17.66 (3.68)	13.09 (4.72)	**< 0.001**
CVLT long delayed Free Recall, mean (SD)	14.13 (1.99)	9.73 (3.64)	**0.002**
Language composite score (z-score), mean (SD)	0.12 (0.37)	−0.43 (0.40)	**< 0.001**
Aβ status, No. (%)			0.35
Negative	55 (69)	6 (55)	
Positive	25 (31)	5 (45)	
*APOE* ε4 status, No. (%)			0.81
Non-carrier	49 (60)	7 (64)	
Carrier	33 (40)	4 (36)	
Left EC SUVR (FreeSurfer)	1.92 (0.81)	2.79 (1.56)	0.10
Right EC SUVR (FreeSurfer)	2.04 (0.90)	2.97 (1.73)	0.11
Left ERC SUVR (ASHS)	1.99 (0.95)	2.81 (1.39)	**0.01**
Right ERC SUVR (ASHS)	2.07 (0.94)	2.88 (1.49)	0.11
Left BA35 SUVR (ASHS)	1.96 (0.92)	2.82 (1.73)	0.13
Right BA35 SUVR (ASHS)	1.92 (0.81)	2.66 (1.09)	**0.008**
Left BA36 SUVR (ASHS)	1.75 (0.40)	2.45 (1.50)	0.15
Right BA36 SUVR (ASHS)	1.78 (0.41)	2.48 (1.30)	0.11

Aβ, amyloid β; APOE, apolipoprotein E; BA, Brodmann area; CDR, Clinical Dementia Rating; CVLT, California Verbal Learning Test; EC/ERC, entorhinal cortex; MCI, Mild Cognitive Impairment; MMSE, Mini-Mental State Examination; SUVR, Standardized Uptake Value Ratio.

Results from the stepwise linear regression analyses are presented in Table 2, and detailed results for each model are described below.

**Table 2:** Stepwise regression models with ^18^F-MK6240 PET explaining variability in composite cognitive measures in right hemisphere models.

Dependent variables	Independent variables	*β*	*t*-value	*F*	*ΔF*	*R^2^*	*ΔR^2^*
CDR-SB							
Step1				19.62***		0.18	
	Right BA36	0.43	4.43***				
Step2				12.89***	5.22*	0.23	0.05
	Right BA36	0.39	4.10***				
	Age	0.22	2.29*				
Step3				10.58***	4.81*	0.27	0.04
	Right BA36	0.68	4.21***				
	Age	0.28	2.84**				
	Right BA35	−0.37	−2.19*				
MMSE							
Step1				9.19**		0.10	
	Right BA36	−0.31	−3.03**				
Step2				7.17***	4.76*	0.14	0.05
	Right BA36	−0.27	−2.70**				
	Age	−0.22	−2.18*				
Delayed memory composite score							
Step1				4.02*	4.02*	0.04	0.04
	Right BA36	−0.21	−2.01*				
Language composite score							
Step1				12.93***		0.13	
	Education	0.36	3.60***				
Step2				11.61***	9.10**	0.21	0.08
	Education	0.35	3.62***				
	Right BA36	−0.29	−3.02**				

Variables entered into each model: age, sex, education, APOE4 carrier status, global amyloid burden, SUVR right EC (FreeSurfer), SUVR right ERC (ASHS), SUVR right BA36 (ASHS), SUVR right BA35 (ASHS). *p < 0.05; **p < 0.01; ***p < 0.001.

### 3.1 CDR Sum of Boxes

**Figure 3:**
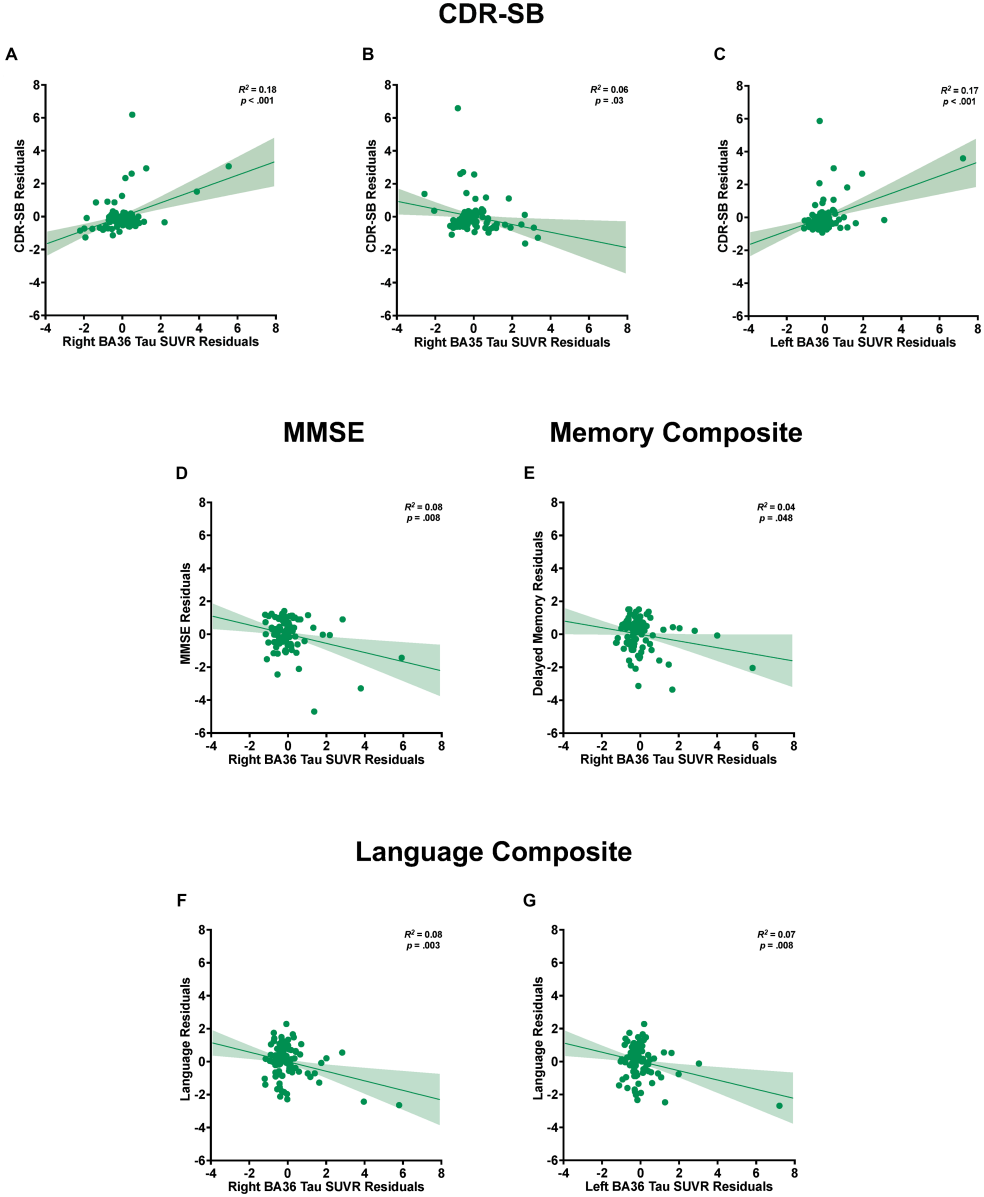
Relationships between tau load in medial temporal lobe (MTL) subregions and cognitive measures. Partial regression plots from stepwise linear regression models with standardized residuals illustrating the association between tau load in MTL subregions and the variability observed in CDR-SB **(A–C)**, MMSE **(D)**, and composite scores of delayed episodic memory **(E)** and language **(F,G)**. The plots include shaded 95% confidence interval bands. Separate models are shown for each dependent measure of cognition and for the left and right hemispheres. Models accounted for potential contributions of age, sex, education, APOE4 status, and global amyloid burden.

**Figure 4:**
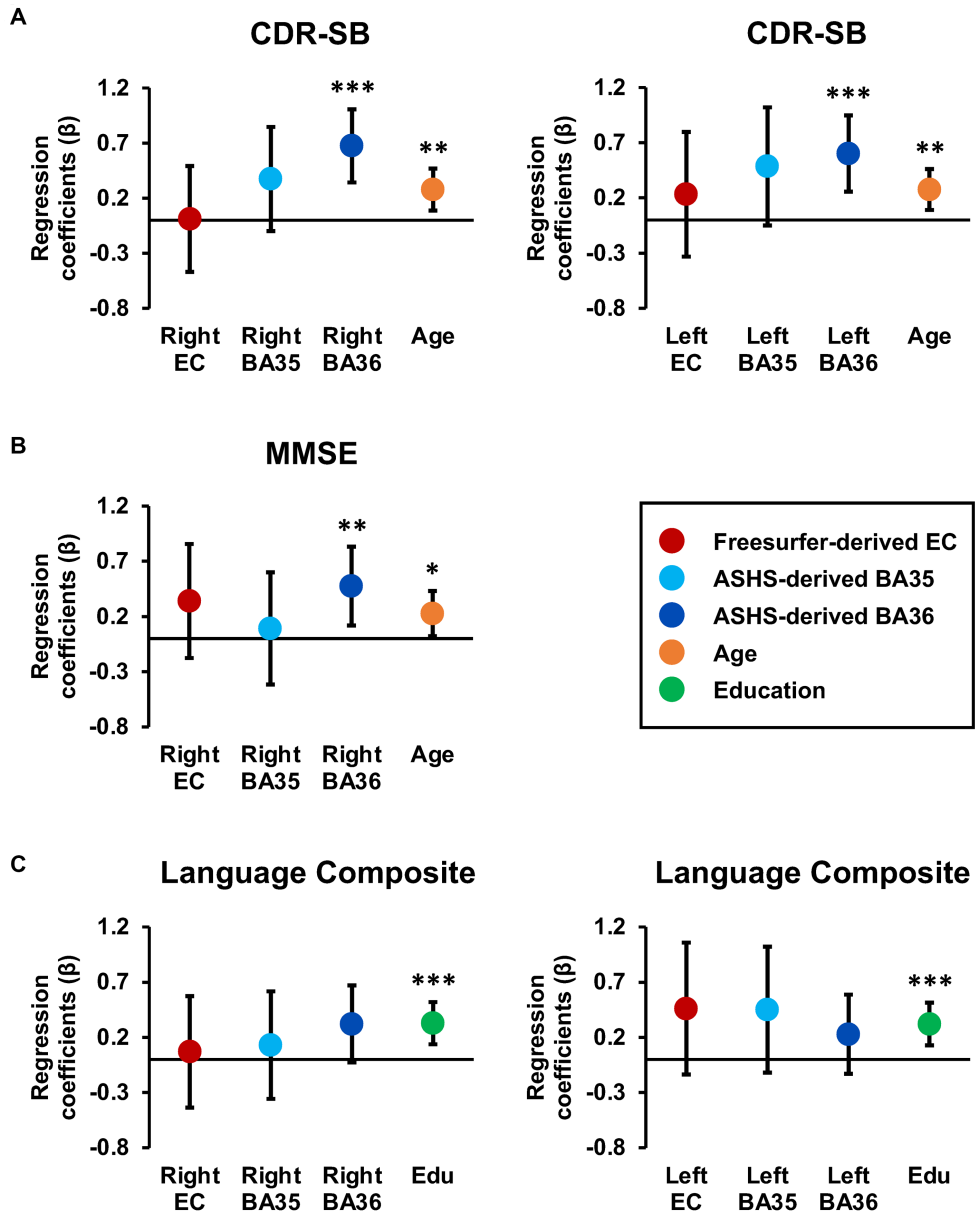
Tau burden in Brodmann area 36 is a significant predictor of CDR-SB and MMSE scores. Regression coefficient betas (absolute values) from the hierarchical regression analyses are plotted for variables of interest, with color coding based on FreeSurfer EC (red), ASHS-generated MTL subregions (light blue and dark blue), age (orange), and education (green) for the CDR-SB **(A)**, MMSE **(B)** and language composite scores **(C)**. The error bars represent 95% confidence intervals. Hierarchical models were constructed using significant demographic predictors from the previous stepwise models, along with the larger inclusive FreeSurfer EC segmentation and the smaller MTL subregion segmentations provided through ASHS. Importantly, tau PET burden in subregions, specifically BA36, consistently emerged as a significant predictor of CDR-SB and MMSE, while the larger FreeSurfer EC label did not emerge as a significant predictor of cognition.

For CDR-SB, the right hemisphere stepwise linear regression model identified right BA36 SUVR [*ß* = 0.68, *t*(86) = 4.21, *p* < 0.001; Figure 3A], age [*ß* = 0.28, *t* (86) = 2.84, *p* = 0.006], and right BA35 SUVR [*ß* = −0.37, *t*(86) = −2.19, *p* = 0.03; Figure 3B] as significant predictors in the final model. These findings indicate that higher tau load in BA36 was associated with increased scores on the CDR-SB, while lower levels of tau load in BA35 were associated with higher CDR-SB scores. Notably, the addition of both of these variables significantly improved the overall proportion of explained variance. Right BA36 tau load was the strongest single predictor [*R^2^* = 0.18, *F*(1,88) = 19.62, *p* < 0.001], with subsequent steps demonstrating significant increases in the proportion of explained variance after adding age in Step 2 [*ΔR^2^* = 0.05, *ΔF*(1,87) = 5.22, *p* = 0.03], and right BA35 in Step 3 [*ΔR^2^* = 0.04, *ΔF* (1,86) = 4.81, *p* = 0.03]. The strongest model incorporated all three variables [*R^2^* = 0.27, *F* (3,86) = 10.58, *p* < 0.001]. Importantly, the remaining variables, including FreeSurfer-derived right EC SUVR, ASHS-derived right ERC SUVR, cerebral cortex amyloid burden, APOE4 status, sex, and education did not significantly contribute to the model (*p’s* > 0.45). Furthermore, a follow-up hierarchical linear regression analysis confirmed that the inclusion of tau load from the FreeSurfer-derived right EC region did not have a significant impact on the proportion of explained variance [*ΔR^2^* = 0.00, *ΔF*(1,88) = 0.002, *p* = 0.96; Figure 4A]. These findings underscore the prominent role of right BA36 tau load, age, and right BA35 tau load in predicting CDR-SB scores, while highlighting the limited contribution of other variables considered in this model.

For the left hemisphere, tau load in left BA36 [*ß* = 0.40, *t* (87) = 4.29, *p* < 0.001; Figure 3C] and age [*ß* = 0.25, *t*(87) = 2.71, *p* = 0.008] were significant predictors of CDR-SB. The positive relationship between tau load in left BA36 and CDR-SB further supports the notion that higher tau load in this region was associated with higher CDR-SB scores. The addition of each variable significantly improved the model, with Step 1 indicating that left BA36 SUVR was a significant predictor [*R^2^* = 0.18, *F*(1,88) = 18.83, *p* < 0.001], followed by a significant increase in the proportion of explained variance after adding age in Step 2 [*ΔR^2^* = 0.06, *ΔF*(1,87) = 7.32, *p* = 0.008]. The strongest model included both variables [*R^2^* = 0.24, *F*(2,87) = 13.75, *p* < 0.001]. No other variables emerged as significant predictors (*p’s* > 0.08). Additionally, the follow-up hierarchical linear regression confirmed that the inclusion of tau load in the left FreeSurfer-derived EC did not significantly change the proportion of explained variance [*ΔR^2^* = 0.006, *ΔF*(1,88) = 0.67, *p* = 0.42; Figure 4A]. Complete model results are shown in Supplementary Table S1.

### 3.2 MMSE

With respect to MMSE, the final stepwise model for the right hemisphere MTL subregions revealed that tau load in right BA36 [*ß* = −0.27, *t*(87) = −2.70, *p* = 0.008; Figure 3D] and age [*ß* = −0.22, *t*(87) = −2.18, *p* = 0.03] were significant predictors. These findings suggest that higher tau load in right BA36 was significantly associated with poorer performance on the MMSE. Each variable significantly improved the model, with Step 1 indicating that tau load in right BA36 was a significant predictor [*R^2^* = 0.10, *F*(1,88) = 9.19, *p* = 0.003], and with the inclusion of age in Step 2 significantly increasing the proportion of explained variance [*ΔR^2^* = 0.05, *ΔF*(1,87) = 4.76, *p* = 0.03]. The strongest model included both variables [*R^2^* = 0.14, *F*(2,87) = 7.17, *p* < 0.001]. Other considered variables, including sex, APOE4 status, cerebral cortex amyloid burden, right FreeSurfer-derived EC SUVR, right ASHS-derived ERC SUVR, and right BA35 SUVR, did not significantly contribute to the model (*p’s* > 0.06). Moreover, in the follow-up hierarchical regression, the inclusion of FreeSurfer-derived right EC SUVR did not significantly alter the proportion of explained variance [*ΔR^2^* = 0.02, *ΔF* (1,88) = 1.72, *p* = 0.19; Figure 4B]. By contrast, the final model for the left hemisphere MTL subregions revealed that age was the only significant predictor [*ß* = −0.26, *t*(88) = −2.57, *p* = 0.01; see Supplementary Table S1], indicating an association between age and individual variation in MMSE scores [*R^2^* = 0.07, *F*(1,88) = 6.58, *p* = 0.01]. No significant associations were observed between MMSE scores and tau PET load in any of the left hemisphere MTL subregions (*p’s* > 0.10).

### 3.3 Delayed episodic memory composite score

For the delayed episodic memory composite score, the final stepwise model for the right hemisphere showed tau load in right BA36 as the only significant predictor [*ß* = −0.21, *t* (88) = −2.01, *p* = 0.048; *R^2^* = 0.04, *F* (1,88) = 4.02, *p* = 0.048; Figure 3E]; the other independent variables did not significantly account for additional variance (*p’s* > 0.51). This finding suggests that increased tau load in right BA36 was associated with poorer delayed episodic memory. Furthermore, in the follow-up hierarchical regression, the inclusion of FreeSurfer-derived right EC SUVR did not significantly change the proportion of explained variance [*ΔR^2^* = 0.007, *ΔF* (1,89) = 0.65, *p* = 0.42]. In the left hemisphere model, no variables emerged as significant predictors of the delayed episodic memory composite score.

### 3.4 Language composite score

For the language composite score, the final stepwise model for the right hemisphere MTL subfields included education [*ß* = 0.35, *t*(87) = 3.62, *p* < 0.001] and right BA36 SUVR [*ß* = −0.29, *t*(87) = −3.02, *p* = 0.003; Figure 3F] as significant predictors. The negative relationship with BA36 indicates that higher tau load in this region corresponded to lower language composite scores. The addition of both variables significantly improved the overall proportion of explained variance in the model, with education emerging as a significant predictor in Step 1 [*R^2^* = 0.13, *F*(1,88) = 12.93, *p* < 0.001]. Subsequently, the addition of tau load in right BA36 in Step 2 resulted in a significant increase in the proportion of explained variance [*ΔR^2^* = 0.08, *ΔF* (1,87) = 9.10, *p* = 0.003]. The strongest model incorporated both [*R^2^* = 0.21, *F*(2,87) = 11.61, *p* < 0.001], while the other considered variables did not significantly contribute to the model (*p’s* > 0.21). Furthermore, in the follow-up hierarchical regression, the addition of FreeSurfer-derived right EC SUVR did not significantly alter the explained variance [*ΔR^2^* = 0.001, *ΔF* (1,88) = 0.07, *p* = 0.79; Figure 4C].

Similarly, the final stepwise model for the left hemisphere MTL subfields showed education [*ß* = 0.36, *t* (87) = 3.78, *p* < 0.001] and tau load in left BA36 were significantly associated with language composite scores [*ß* = −0.26, *t* (87) = −2.74, *p* = 0.008; Figure 3G]. Once again, the addition of each variable significantly improved the predictability of the overall model, with education included in Step 1 [*R^2^* = 0.13, *F* (1,88) = 12.93, *p* < 0.001], and the addition of right BA36 tau load in Step 2 [*ΔR^2^* = 0.07, *ΔF* (1,87) = 7.48, *p* = 0.008]. The strongest model included both variables [*R^2^* = 0.20, *F*(2,87) = 10.68, *p* < 0.001], and no other variables reached significance for inclusion (*p’s* > 0.15). The follow-up hierarchical regression analysis confirmed that the addition of FreeSurfer-derived left EC tau load did not significantly enhance the proportion of explained variance in the final model [*ΔR^2^* = 0.02, *ΔF*(1,88) = 2.36, *p* = 0.13; Figure 4C]. These findings emphasize the significant role of education and tau load in BA36 in predicting the language composite score, while highlighting the limited contribution of other regions and demographic variables. Detailed results are shown in Supplementary Table S1.

### 3.5 Sensitivity analyses

To test whether our findings were primarily driven by a subgroup of participants, we performed follow-up sensitivity analyses where stepwise regression analyses were computed separately for each diagnostic group. In the subsample of CN individuals only, right BA35 SUVR [*ß* = 0.99, *t* (76) = 3.07, *p* = 0.003] and right ASHS-derived ERC SUVR [*ß* = −0.80, *t* (76) = −2.48, *p* = 0.02] were significant predictors of CDR-SB. For the left hemisphere model, left FreeSurfer-derived EC tau load significantly contributed to the proportion of explained variance in CDR-SB [*ß* = 0.24, *t* (77) = 2.19, *p* = 0.03]. No significant associations were found for MMSE models in the CN only subsample. For both right and left hemisphere models, age was the only significant predictor to emerge from the delayed episodic memory composite models [*ß* = 0.23, *t* (77) = 2.05, *p* = 0.04]. Similarly, for both right and left hemisphere models, education was the only significant predictor of language composite scores [*ß* = 0.41, *t* (77) = 3.97, *p* < 0.001]. Detailed results are shown in Supplementary Table S2.

In the subsample of MCI patients only, no significant predictors were found in stepwise models for either hemisphere predicting CDR-SB or delayed episodic memory composite. For both right and left hemisphere models, sex significantly contributed to the proportion of explained variance in MMSE scores [*ß* = 0.75, *t* (9) = 3.42, *p* < 0.008]. Finally, right BA36 SUVR was significantly associated with language composite scores in the MCI only subsample [*ß* = −0.71, *t*(9) = −2.98, *p* = 0.02]. It is important to note the exploratory nature of the MCI only subgroup analysis, as it is underpowered due to the very small number of MCI patients in this study. The overall pattern of results from these sensitivity analyses is consistent with the overall findings and further supports the utility of MTL subregional analyses, as subtle differences in patterns of tau accumulation may emerge over time or across individuals. Complete results are shown in Supplementary Table S3.

## 4 Discussion

This study evaluated contributions of regional tau burden in specific MTL subregions, as measured by ^18^F-MK6240 PET, to individual variability in cognition in order to determine whether tau burden localized to subregions of the entorhinal cortex provides additional specificity compared to tau burden quantified in larger segmentations. Taking advantage of the high binding specificity of this second-generation tracer, in combination with the application of advanced registration and segmentation methods provided by the ANTS and ASHS software packages, this approach enabled quantification of *in vivo* tau burden within specific subregions of the MTL. Results showed that tau burden, specifically in left and right BA36 and right BA35, as well as age, were each independently related to individual differences in CDR-SB scores. Similarly, tau burden in right BA36 and age each uniquely contributed to the proportion of explained variance in MMSE scores, while left and right BA36 tau burden and education were associated with language composite scores, and right BA36 tau burden significantly predicted delayed episodic memory composite scores.

While the stepwise regression analyses aimed to identify the best overall combination of variables to explain individual differences in cognition, irrespective of the origin of the ROIs (i.e., FreeSurfer vs. ASHS), we were also interested in directly comparing the relative contributions of the FreeSurfer EC ROI to the ASHS MTL ROIs. To address this question, hierarchical regression analyses were used to ensure that the FreeSurfer EC ROI was retained in the models regardless of the predictor’s statistical significance. Importantly, the addition of the more inclusive ROI encompassing a broader parcellation of the entorhinal cortex did not significantly contribute to the explained variance in any of the cognitive measures. Together, these findings suggest that quantification of tau burden within more localized subregions of the MTL may better account for individual differences in cognition in non-demented older adults.

These findings are not only consistent with previous reports showing significantly increased accumulation of tau deposition in MTL subregions using ^18^F-AV1451 PET imaging (Johnson et al., 2016), but further localize early deposition of tau to the right BA35 subregion. BA35 largely overlaps with what Braak and colleagues referred to as TEC, where they observed some of the earliest neurofibrillary tangle accumulation in patients with MCI due to AD (Braak and Braak, 1990; Braak et al., 2006; Kaufman et al., 2018). The results further demonstrate significant relationships between tau burden in MTL subregions and cognition in non-demented older adults. Specifically, tau burden in BA36 was associated with individual differences across all cognitive assessments, indicating the importance of this region in the interaction between pathological tau accumulation and cognition.

Additionally, there was a significant association between BA35 and CDR-SB scores (a measure designed to reflect both cognition and function). It is important to note that the direction of this relationship showed that lower levels of tau load in BA35 were associated with higher CDR-SB scores. Given the strong positive association between tau load in BA36 and CDR-SB, this negative relationship with tau load in BA35 likely reflects the topographical progression of tau accumulation with disease progression described in Braak staging.

The observed relationship between tau burden and memory aligns with the functional role of the brain regions where this effect was observed. The temporal lobe plays a critical role in episodic memory function but also plays an important role in language function, so it is possible that the language finding in our data may reflect some interplay between different temporal lobe regions and cognitive functions. Although few studies have examined subregion-specific tau pathology accumulation using PET imaging (Adams et al., 2019; Berron et al., 2021; Chen et al., 2021; Ge et al., 2021), functional specialization of MTL subregions has been previously reported using functional MRI (Maass et al., 2015; Adams et al., 2021). Studies of CN adults have shown that the lateral EC and perirhinal cortex support encoding of object and content information, while medial EC and parahippocampal regions are thought to facilitate encoding of context and spatial information (Knierim et al., 2014; Reagh and Yassa, 2014; Yeung et al., 2017; Berron et al., 2018; Montchal et al., 2019).

Similarly, studies of patients with MCI have shown reduced volume in the lateral EC, as well as reduced activation in the lateral EC, when compared to CN participants (Reagh et al., 2018; Tran et al., 2021, 2022). These changes in the lateral EC are associated with the memory performance of individuals with MCI, which are not observed in association with the medial EC. These findings are consistent with the results reported here, as lateral EC overlaps primarily with BA35 and partially with BA36, where associations with tau and cognition were observed, while the medial EC corresponds to the ERC label, where such associations were not present. Given the observed functional specialization and selective engagement of EC subregions in patients with MCI, the examination of tau burden in more localized subregions of the MTL may provide new opportunities to understand individual differences and improve the identification of non-demented older adults who are on a trajectory of decline due to AD. However, it is important to note that this study measured tau SUVR in MTL regions, rather than functional activation, cortical thickness, or volume; as such, it is not necessarily expected to see cognitive domain-specific effects associated with tau burden. Given the established association between the progression of tau accumulation and overall decline in cognition throughout disease progression, it seems reasonable that individual differences in tau burden in these early Braak regions would be associated with individual differences in overall cognition.

Several limitations of the current study should be noted. First, the ROIs used in the current study compared the FreeSurfer and ASHS softwares, which each employ somewhat different boundaries for the EC with none of the segmented regions in either software being fully consistent with the TEC used in the post-mortem studies showing the earliest stages of tau. Functional studies of the EC have employed a lateral versus medial distinction; however, those boundaries are similarly not consistent across studies. A consensus of landmarks and terminology based on both anatomical and functional studies is needed to facilitate comparisons across studies and further assess the functional correlates of tau accumulation in subregions of the EC. Second, the present analyses are cross-sectional, based on a single assessment of tau load, limiting claims with respect to changes in tau spread and cognition over time. However, data collection in this cohort is ongoing, making longitudinal analyses possible in future studies. Finally, it is important to note that the study only included a small number of MCI patients, limiting our ability to detect potential group differences or make claims regarding the MCI subgroup. To determine whether the pattern of results was driven by this small group of MCI patients, a series of follow-up sensitivity analyses was performed limiting our stepwise regressions to include only CN individuals or only MCI individuals. Importantly, the overall pattern of results within the CN subgroup was consistent with the primary findings, indicating that the relationships between tau burden in MTL subregions and cognition were not solely driven by the MCI subgroup of patients. In these constrained analyses, the localized subregions remained informative, supporting the conclusion that consideration of specific subregions may enhance sensitivity for the identification of individuals on the AD-trajectory.

## 5 Conclusion

The current study examined associations between regional tau burden in specific MTL subregions and relationships with individual differences in cognition in non-demented older adults. The findings indicate that applying advanced registration and segmentation methods to tau PET images can achieve enhanced visualization and quantification of tau uptake localized to subregions within the MTL. Estimates of tau load in specific MTL subregions furthermore explain variance in individual differences in CDR-SB scores and across multiple cognitive domains. Together, these findings suggest that tau accumulation localized to subregions of the entorhinal cortex provides additional specificity compared to accumulation quantified in larger segmentations, therefore highlighting the importance of examining associations between localized tau burden and cognitive variability for improving the characterization of individual disease trajectories and identifying those individuals who are on a trajectory of decline due to AD.

## Data availability statement

The raw data supporting the conclusions of this article will be made available by the authors, without undue reservation.

## Ethics statement

The studies involving humans were approved by Johns Hopkins University Institutional Review Board. The studies were conducted in accordance with the local legislation and institutional requirements. The participants provided their written informed consent to participate in this study.

## Author contributions

NR: Data curation, Formal analysis, Methodology, Visualization, Writing – original draft, Writing – review & editing. KA: Formal analysis, Methodology, Visualization, Writing – review & editing, Data curation, Writing – original draft. CC-L: Methodology, Writing – review & editing. CS: Writing – review & editing, Data curation, Validation. AS: Conceptualization, Data curation, Methodology, Project administration, Writing – review & editing. CP: Conceptualization, Data curation, Methodology, Project administration, Writing – review & editing. YZ: Methodology, Writing – review & editing. MA: Conceptualization, Data curation, Funding acquisition, Investigation, Methodology, Project administration, Resources, Supervision, Validation, Writing – review & editing. AB: Conceptualization, Data curation, Funding acquisition, Investigation, Methodology, Project administration, Resources, Supervision, Validation, Writing – review & editing.
