# Supplementary material for: Tau PET burden in Brodmann areas 35 and 36 is associated with individual differences in cognition in non-demented older adults
Source: Front Aging Neurosci. 2023 Dec 14;15:1272946. doi: 10.3389/fnagi.2023.1272946 (PMC10757623; doi:10.3389/fnagi.2023.1272946)
Supplement: Supplementary file 1 [file Data_Sheet_1.pdf]

Supplementary Material

Tau PET Burden in Brodmann areas 35 and 36 is associated with individual differences in cognition in non-demented older adults

Nisha Rani[1†], Kylie H. Alm[1†], Caitlin A. Corona-Long[1], Caroline L. Speck[1], Anja Soldan[2], Corinne Pettigrew[2], Yuxin Zhu[2], Marilyn Albert[2], Arnold Bakker[1,2*]

† These authors contributed equally to this work and share first authorship.

*Corresponding Author:
Arnold Bakker, PhD
Email: abakker@jhu.edu

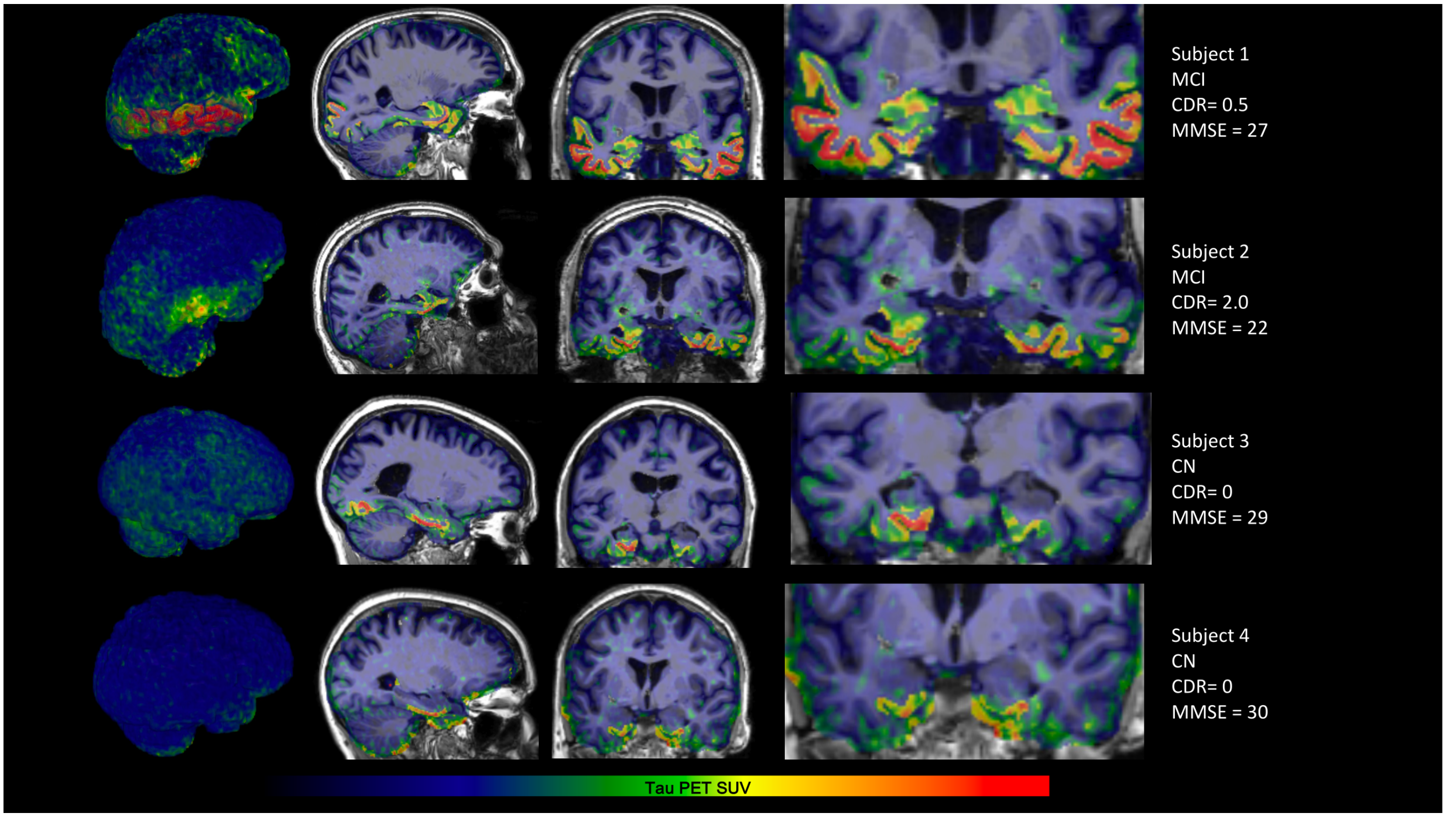

Supplementary Figure 1. Representative examples of ^{18}F-MK6240 PET images registered to the subject's T1-weighted MRI, showcasing the PET-MRI registration and the distribution of the tracer in both MCI subjects (top two rows) and cognitively normal (CN) individuals (bottom two rows).

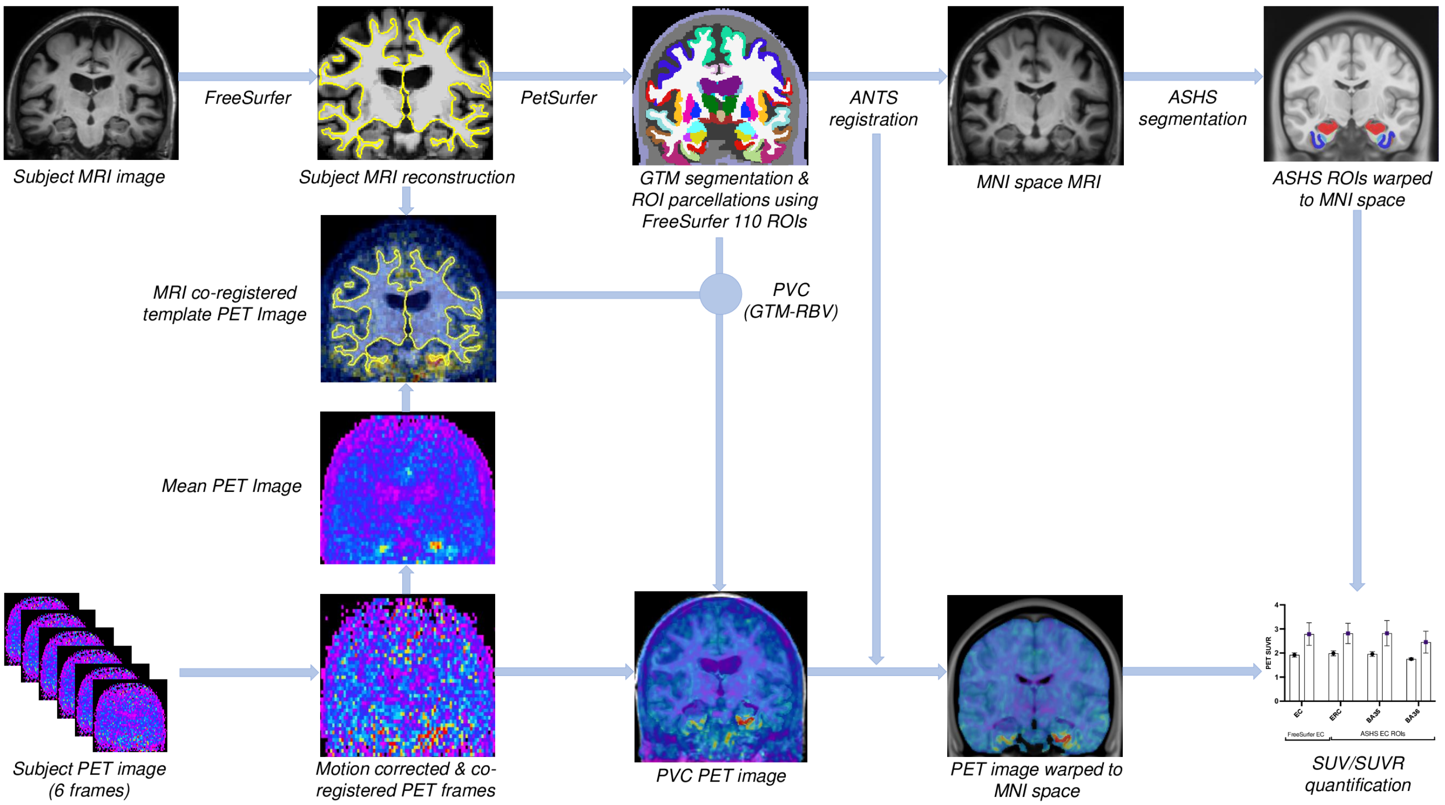

Supplementary Figure 2. PET image analysis pipeline steps.

Supplementary Table 1. Stepwise regression models with ^{18}F-MK6240 PET explaining variability in composite cognitive measures in left hemisphere models.

**Dependent variables**	**Independent variables**	***β***	***t-value***	***F***	***ΔF***	***R²***	***ΔR²***
**CDR-SB**							
Step1				18.83***		0.18	
	Left BA36	0.42	4.34***				
Step2				13.75***	7.32*	0.24	0.06
	Left BA36	0.40	4.29***				
	Age	0.25	2.71**				
**MMSE**							
Step1				6.58*		0.07	
	Age	-0.26	-2.57*				
**Language composite score**							
Step1				12.93***		0.13	
	Education	0.36	3.60***				
Step2				10.68***	7.47**	0.20	0.07
	Education	0.36	3.78***				
	Left BA36	-0.26	-2.74**				

*Variables entered into each model: age, sex, education, APOE4 carrier status, global amyloid burden, SUVR left EC (FreeSurfer), SUVR left ERC (ASHS), SUVR left BA36 (ASHS), SUVR left BA35 (ASHS). *p < .05; **p < .01; ***p < .001.*

Supplementary Table 2. Stepwise regression models with ^{18}F-MK6240 PET explaining variability in composite cognitive measures within cognitively normal participants only (N = 82).

Dependent variables	Independent variables	β	*t*-value	F	ΔF	R^2	ΔR^2
**CDR-SB**							
Step1				4.79*		0.06	
	Left EC	0.24	2.19*				
**CDR-SB**							
Step1				4.56*		0.06	
	Right BA35	0.24	2.14*				
Step2				5.50**	6.14*	0.13	0.07
	Right BA35	0.99	3.07**				
	Right ERC	-0.80	-2.48*				
**Delayed memory composite score**							
Step1				4.20*		0.05	
	Age	0.23	2.05*				
**Language composite score**							
Step1				15.78***		0.17	
	Education	0.41	3.97***				

*Variables entered into each model: age, sex, education, APOE4 carrier status, global amyloid burden, SUVR EC (FreeSurfer), SUVR ERC (ASHS), SUVR BA36 (ASHS), SUVR BA35 (ASHS). *p < .05; **p < .01; ***p < .001.*

Supplementary Table 3. Stepwise regression models with ^{18}F-MK6240 PET explaining variability in composite cognitive measures within mild cognitive impairment participants only (N = 11).

**Dependent variables**	**Independent variables**	β	***t*-value**	***F***	ΔF	R^2	ΔR^2
**MMSE**							
Step1				11.66**		0.56	
	Sex	0.75	3.42**				
**Language composite score**							
Step1				8.90*		0.50	
	Right BA36	-0.71	-2.98*				

*Variables entered into each model: age, sex, education, APOE4 carrier status, global amyloid burden, SUVR EC (FreeSurfer), SUVR ERC (ASHS), SUVR BA36 (ASHS), SUVR BA35 (ASHS). *p < .05; **p < .01; ***p < .001.*
